# Nine-gene signature and nomogram for predicting survival in patients with head and neck squamous cell carcinoma

**DOI:** 10.3389/fgene.2022.927614

**Published:** 2022-08-24

**Authors:** Fan Yang, Liu-qing Zhou, Hui-wen Yang, Yan-jun Wang

**Affiliations:** Department of Otorhinolaryngology, Union Hospital, Tongji Medical College, Huazhong University of Science and Technology, Wuhan, China

**Keywords:** head and neck squamous cell carcinoma, TCGA, GEO, prognosis, gene signature, bioinformatics analysis

## Abstract

**Background:** Head and neck squamous cell carcinomas (HNSCCs) are derived from the mucosal linings of the upper aerodigestive tract, salivary glands, thyroid, oropharynx, larynx, and hypopharynx. The present study aimed to identify the novel genes and pathways underlying HNSCC. Despite the advances in HNSCC research, diagnosis, and treatment, its incidence continues to rise, and the mortality of advanced HNSCC is expected to increase by 50%. Therefore, there is an urgent need for effective biomarkers to predict HNSCC patients’ prognosis and provide guidance to the personalized treatment.

**Methods:** Both HNSCC clinical and gene expression data were abstracted from The Cancer Genome Atlas (TCGA) database. Intersecting analysis was adopted between the gene expression matrix of HNSCC patients from TCGA database to extract TME-related genes. Differential gene expression analysis between HNSCC tissue samples and normal tissue samples was performed by R software. Then, HNSCC patients were categorized into clusters 1 and 2 via NMF. Next, TME-related prognosis genes (*p* < 0.05) were analyzed by univariate Cox regression analysis, LASSO Cox regression analysis, and multivariate Cox regression analysis. Finally, nine genes were selected to construct a prognostic risk model and a prognostic gene signature. We also established a nomogram using relevant clinical parameters and a risk score. The Kaplan–Meier curve, survival analysis, time-dependent receiver operating characteristic (ROC) analysis, decision curve analysis (DCA), and the concordance index (C-index) were carried out to assess the accuracy of the prognostic risk model and nomogram. Potential molecular mechanisms were revealed by gene set enrichment analysis (GSEA). Additionally, gene correlation analysis and immune cell correlation analysis were conducted for further enriching our results.

**Results:** A novel HNSCC prognostic model was established based on the nine genes (GTSE1, LRRN4CL, CRYAB, SHOX2, ASNS, KRT23, ANGPT2, HOXA9, and CARD11). The value of area under the ROC curves (AUCs) (0.769, 0.841, and 0.816) in TCGA whole set showed that the model effectively predicted the 1-, 3-, and 5-year overall survival (OS). Results of the Cox regression assessment confirmed the nine-gene signature as a reliable independent prognostic factor in HNSCC patients. The prognostic nomogram developed using multivariate Cox regression analysis showed a superior C-index over other clinical signatures. Also, the calibration curve had a high level of concordance between estimated OS and the observed OS. This showed that its clinical net can precisely estimate the one-, three-, and five-year OS in HNSCC patients. The gene set enrichment analysis (GSEA) to some extent revealed the immune- and tumor-linked cascades.

**Conclusion:** In conclusion, the TME-related nine-gene signature and nomogram can effectively improve the estimation of prognosis in patients with HNSCC.

## Background

Globally, head and neck squamous cell carcinoma (HNSCC) is the sixth commonest cancer (Yang et al., 2017). HNSCC is the most common pathological type of head and neck tumors, accounting for over 90% of the cases (Shield et al., 2017). Despite advances in early diagnosis and multi-disciplinary management of cancer, the 5-year overall survival (OS) of HNSCC remains at 50% (González-Arriagada et al., 2018). Results of various studies have shown that gene signatures can predict the prognosis of patients with HNSCC. Multiview data, generated through data integration, are receiving more attention from scholars (Ge et al., 2017). However, gene expression datasets are characterized by high dimensionality, small sample sizes, and sample imbalance. Non-negative matrix factorization (NMF) is frequently used in data analysis to reduce dimension. Its applications in the analysis of gene expression data include sample clustering and feature selection (Liu et al., 2018). The tumor microenvironment (TME) is mainly composed of tumor cells and tumor-invading immune cells admixed with the stromal component. The TME of HNSCC harbors transformed cells, immune cells, and stromal cellular elements (Puram et al., 2017). The TME can have adverse or beneficial consequences. The TME can promote tumor growth and progression through the immune cells which are affected easily (Kim and Bae, 2016). The immune invasive landscape of HNSCC has been elucidated in a previous study (Zhang et al., 2020). A separate study has also performed a systematic analysis of cellular interactions in TME of HNSCC (Huo et al., 2020). In the present study, gene expression profiles of HNSCC TME-related genes were obtained and utilized to create a robust prognostic model and establish a prognostic nomogram.

## Materials and methods

### Sample datasets

We used The Cancer Genome Atlas (TCGA) database (https://cancergenome.nih.gov/) to retrieve HNSCC gene expression data and clinical data. Finally, we downloaded 213 samples (199 HNSCC tissue samples and 14 normal tissue samples) of HNSCC gene expression data. TCGA HNSCC clinical data of 511 patients were also downloaded. The clinical characteristics, including survival status, survival outcomes, age, gender, grade, TNM classification, and stage, were all collected. The GSE16076 dataset with 270 samples (including gene expression data and clinical data) from the Gene Expression Omnibus (GEO) database was downloaded to externally validate the reliability of the nine-gene signature.

### Identification of TME-related genes

First, intersecting analysis was adopted between the gene expression matrix of HNSCC patients from TCGA database to extract TME-related genes. Then, differential expression analysis was performed to filter differentially expressed TME-related genes between HNSCC tissue samples and normal tissue samples by using the “limma” R package. The volcano plot and heatmap were generated to visualize the distribution of the identified TME-related genes by “ggplot2” and “pheatmap” R packages, respectively.

### Identification and evaluation of subgroups

After differential expression analysis, NMF clustering algorithm was applied to the TME-related genes to classify HNSCC patients using the “NMF” R package. An elementary classification of HNSCC patient subgroups was set from 2 to 10. The optimal value for consensus clustering was identified as 2 by the NMF rank survey. Then, the consensus heatmap was generated to view the distribution characteristics among C1 and C2 groups. The Kaplan–Meier curves were applied to explore the discrimination between C1 and C2 groups in HNSCC patients’ OS using “survival” and “survminer” R packages. The statistical difference was evaluated by the log-rank test. In addition, we also adopted the MCP-counter algorithm to assess the difference of tumor-infiltrating immune cells between different clusters (Becht et al., 2016).

### Construction and validation of the prognostic risk model

Differentially expressed TME-related genes between the HNSCC tissue samples and normal tissue samples were abstracted from the previously downloaded TCGA HNSCC gene expression data. From this process, we removed six HNSCC tumor tissue samples. Then, we combined TCGA-HNSCC gene expression data along with clinical data using the “limma” R package. Patients with incomplete clinical data were excluded. Based on previous efforts, a list of 193 TME-related tumor samples with essential clinical information and an OS of >1 month was extracted. Next, total samples from TCGA database were split randomly into the training set (n = 137) and the testing set (n = 56). The training set was applied to determine the TME-related genes as prognostic biomarkers along with the signature. Univariate COX regression analysis, LASSO Cox regression analysis, and multivariate Cox regression analysis were conducted in this process using the “survival” and “glmnet” R packages. Ultimately, nine genes were recognized as prognostic biomarkers to construct the prognostic risk model. The risk score for each HNSCC patient was determined using the same predictive gene signature-based approach. TCGA testing set was used for internal validation for the nine-gene signature. The time-dependent receiver operating characteristic (ROC) analysis and Kaplan–Meier assessments were then used to test the prognostic gene signature using “timeROC” and “survival” R packages. Furthermore, the Wilcoxon signed-rank test was adopted to compare HNSCC tissue samples with normal tissue samples. Statistical significance was set at *p* < 0.05.

### Construction and verification of a predictive nomogram

Nomograms are visual statistical tools that combine multiple clinical and pathological factors to estimate the survival outcomes of patients with cancer (Iasonos et al., 2008). The independent clinical parameters were merged into a nomogram to estimate 1-, 3-, and 5-year survival of patients with HNSCC. Cox regression analysis was conducted to analyze the retrieved clinical parameters from TCGA-HNSCC clinical data using the “survival” R package. Clinical parameters (*p* < 0.05) significantly related to OS were used to establish the predictive nomogram using “regplot” and “rms” R packages. The nomogram was then verified based on the discrimination along with calibration curves. The concordance index (C-index) of the nomogram was determined for each independent clinical parameter and risk score to evaluate the nomogram preceding others through a bootstrap approach with 1,000 resamples. Decision curve analysis (DCA) was applied to determine the clinical net benefit of each clinical parameter and showed that the nomogram harbored the highest net benefit, followed by the risk model. Generally, the nomogram was significantly superior at predicting clinical outcomes (Vickers and Elkin, 2006).

### Gene set enrichment analysis

Gene set enrichment analysis (GSEA) was performed using KEGG (Kyoto Encyclopedia of Genes and Genomes) pathway enrichment analysis. Furthermore, Gene Ontology (GO) functional annotation was used to identify the active genes in various groups of the predictive gene signature in the present study (Subramanian et al., 2005). The differentially expressed TME-related genes were stratified into the high- and low-risk groups based on the median risk score using the “clusterProfiler” R package. The low- and high-risk groups were then subjected to GSEA using the “enrichplot” R package. The statistical significance function was indicated by *p* < 0.05.

### Differentiating performance of the prognostic risk model

The present study used survival analysis to assess the differential diagnostic potential of the risk score. Patients were stratified by stage, grade, age, and gender. Then, we conducted subgroup analysis to confirm the prognostic significance of the risk model in different subgroups.

### External validation of the nine-gene signature

We carefully retrieved and selected three previously established prognostic risk models from PubMed and obtained the relevant genes contained in each model (Wang et al., 2020; Yao et al., 2020; Xiong et al., 2021). The included gene signatures were all validated as reliable prognostic models for forecasting the HNSCC patients’ OS. Then, the Kaplan–Meier curve, time-independent ROC analysis, and the C-index analysis were used to confirm the advantages of the prognostic model established by us. In addition, we downloaded an independent dataset, GSM16076, to further validate the nine-gene signature. The obtained results showed that the prognostic model of the current study is significantly reliable.

### Correlation analysis

Using the “corrplot” R package, immune cell correlation analysis was conducted for further enriching our results. Furthermore, we also conducted Spearman correlation analysis using “corrplot” R package to demonstrate the relationship between risk score and genes.

## Results

### Identification of TME-related genes

**FIGURE 1:**
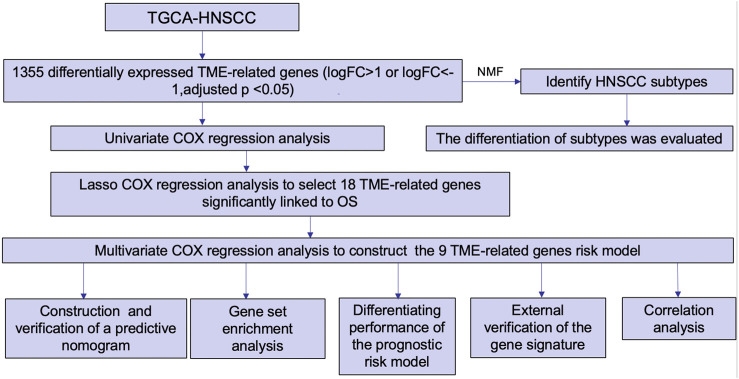
Flow chart of the present study.

**FIGURE 2:**
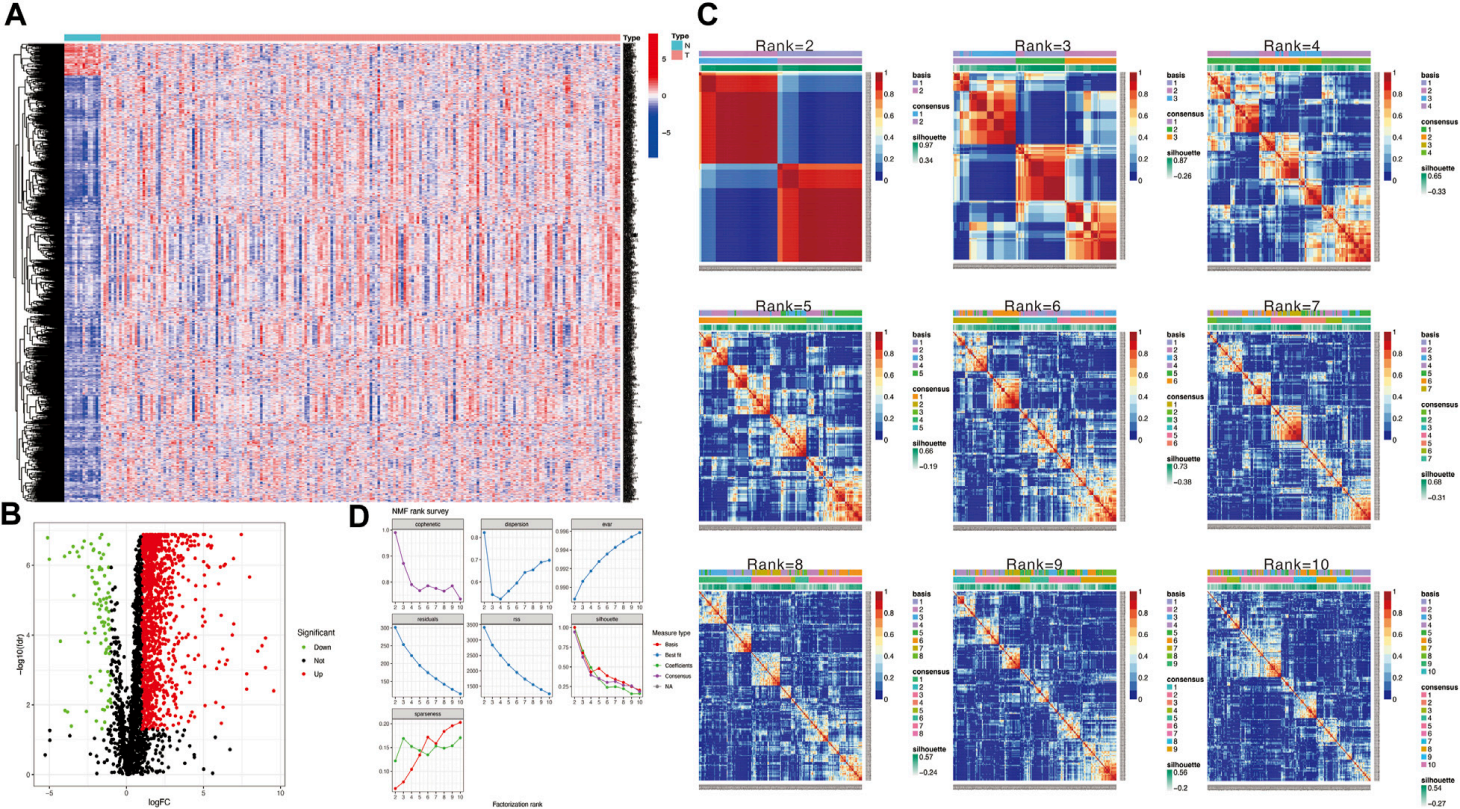
**(A)** Heatmap to show TME-related differentially expressed genes between HNSCC and normal tissue samples. **(B)** Volcano plot displays 1,335 TME-related differentially expressed genes in TCGA HNSCC cohort. **(C)** Non-negative matrix factorization (NMF) clustering was conducted to classify HNSCC patients into 2 to 10 different subtypes, and relevant heatmaps were generated. **(D)** NMF rank survey with multiple parameters (including dispersion, cophenetic, residuals, evar, rss, and silhouette coefficients) to determine the optimal value for consensus clustering.

The flow chart in Figure 1 presents the analytical process of this study. Analysis of the data of 213 samples (199 HNSCC tumor tissue samples and 14 normal tissue samples) obtained from TCGA database was performed. Based on the gene expression levels in TME of the 213 TCGA HNSCC samples, the TME-related genes were selected (*p* < 0.05). After differential expression analysis, a total of 1,335 genes were identified as differentially expressed TME-related genes (Figure 2A). The volcano plot was applied to visualize the upregulated and downregulated genes (Figure 2B).

### Identification and evaluation of subgroups

**FIGURE 3:**
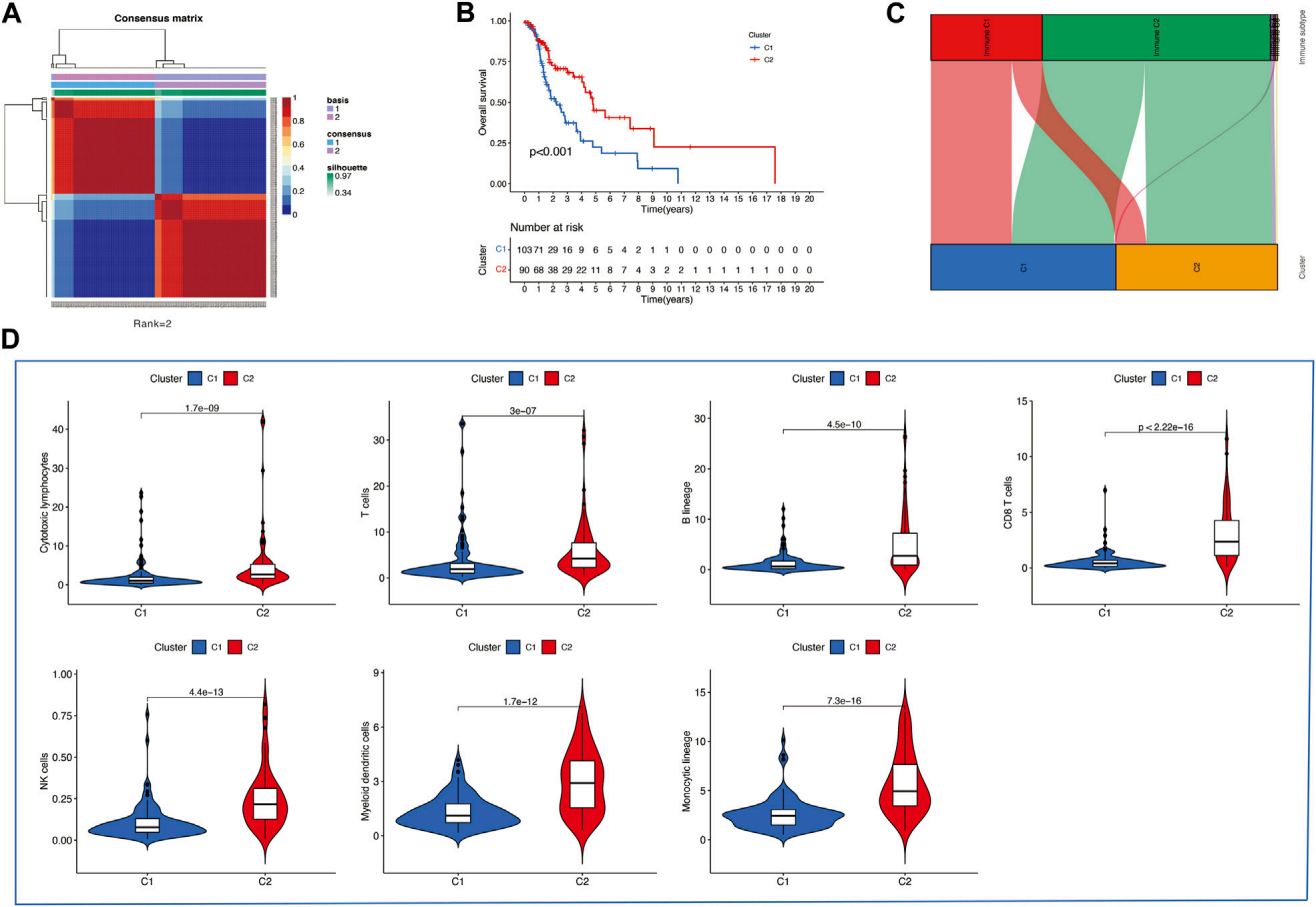
**(A)** Consensus map of the NMF clustering data of TCGA HNSCC cohort. Rank = 2 means that HNSCC patients were separated into two groups. **(B)** Survival curve of OS (P=<0.001) in cluster 1 and 2. **(C)** Sankey plot to show the association between different subtypes and immune subtypes. **(D)** Differential analysis of tumor-infiltrating immune cells was conducted using MCP-counter.

Then, we applied the NMF clustering algorithm method based on the 1,335 identified TME-related genes. Patients were first classified into 2 to 10 different subtypes, and relevant heatmaps were generated (Figure 2C). According to the parameters such as cophenetic, dispersion, silhouette, and sparseness in the NMF rank survey, the optimal number of the cluster was identified as 2 (Figure 2D). HNSCC patients were divided into two subgroups (cluster 1 and cluster 2) (Figure 3A). OS was compared between clusters 1 and 1 to further understand the clustering finding and its links with survival outcomes. According to this assessment, patients in cluster 2 had a superior OS to those in cluster 1 (*p* < 0.001, Figure 3B). The Sankey plot could also be applied to reveal the association between different immune subtypes and clusters (Figure 3C). In addition, the MCP-counter algorithm was used to evaluate the infiltration of the immune cells in cluster 1 and cluster 2. The results revealed that cluster 2 had a higher infiltration level of cytotoxic lymphocytes, T cells, B lineage, CD8^+^ T cells, NK cells, myeloid dendritic cells, and monocytic lineages than cluster 1 (Figure 3D).

### Construction and internal validation of the nine-gene signature

**FIGURE 4:**
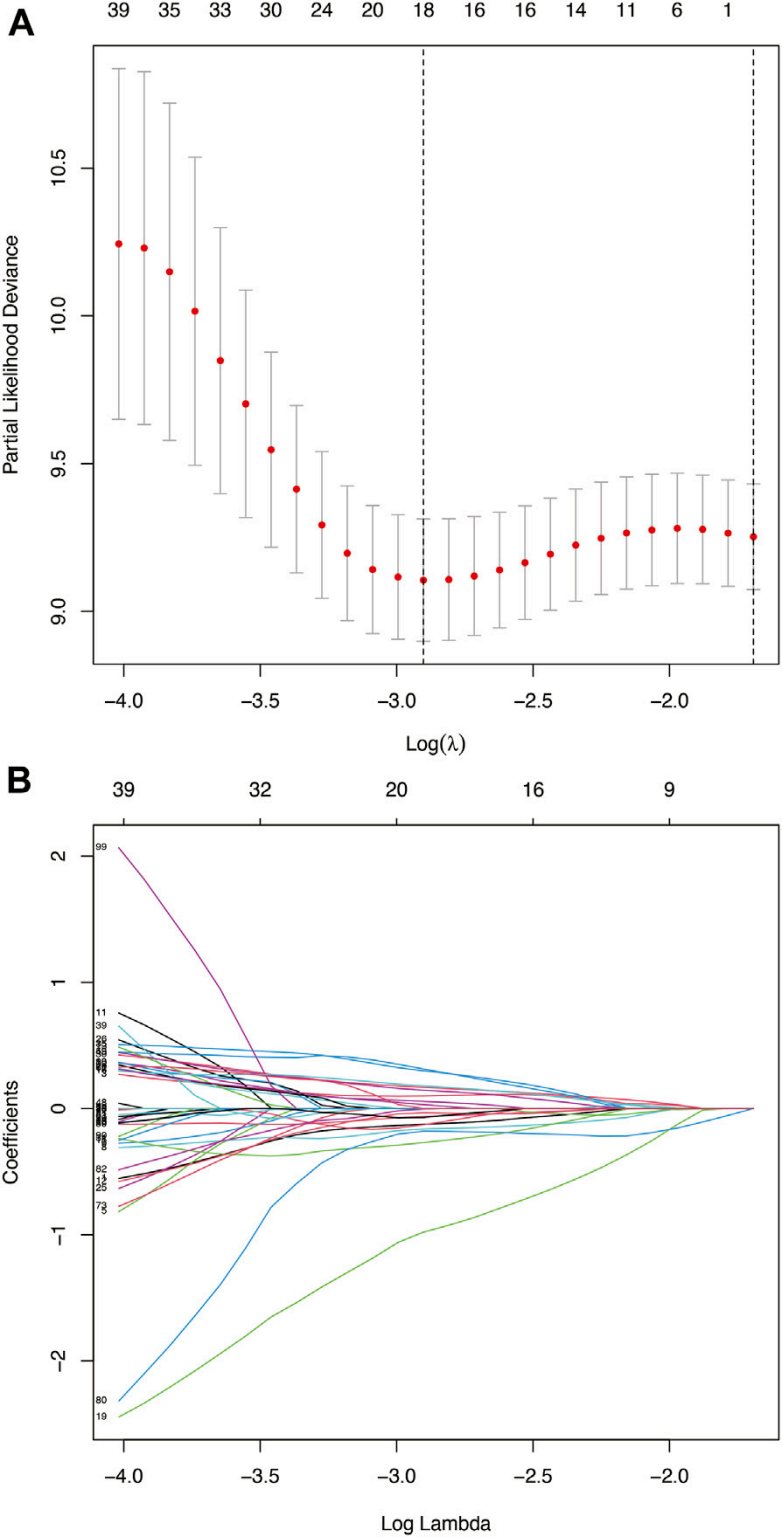
**(A)** Deviance plot of partial likelihood by LASSO Cox regression analysis. Red dots: the partial likelihood of deviance values; gray lines: the standard error (SE); vertical dotted line on the left: the optimal values by minimum criteria; vertical dotted line on the right: 1−SE criteria. **(B)** LASSO coefficient profiles of the 121 TME-related genes in HNSCC.

A total of 193 patients (including 1,335 TME-related genes) included in the survival analysis underwent a follow-up exceeding one month and were randomly assigned into the training set (n = 137) and testing set (n = 56). Data from the training set were retrieved using the “survival” R package and subjected to univariate Cox regression analysis. Subsequently, 121 TME-related genes were selected based on a *p* < 0.05 cut-off value for LASSO Cox regression analysis (Figure 4A). Results of the LASSO Cox regression analysis detected 18 TME-related genes. Then, using multivariate Cox regression analysis, nine genes (GTSE1, LRRN4CL, CRYAB, SHOX2, ASNS, KRT23, ANGPT2, HOXA9, and CARD11) were determined to establish the prognostic risk model. The nine-gene signature as a prognostic marker was an independent prognostic factor tested by the multivariate Cox regression analysis. Significance was defined as *p* < 0.05. Next, nine genes and their corresponding regression coefficients were used to calculate risk scores for each sample using the following formula: risk score = sum of each gene’s (regression coefficient × gene expression value). The optimal risk score cutoff was calculated from the median risk score using the “survminer” R package in the training set. Patients with risk scores above the median scores were assigned to the high-risk group, whereas those with scores below the median risk score were assigned to the low-risk group.

**FIGURE 5:**
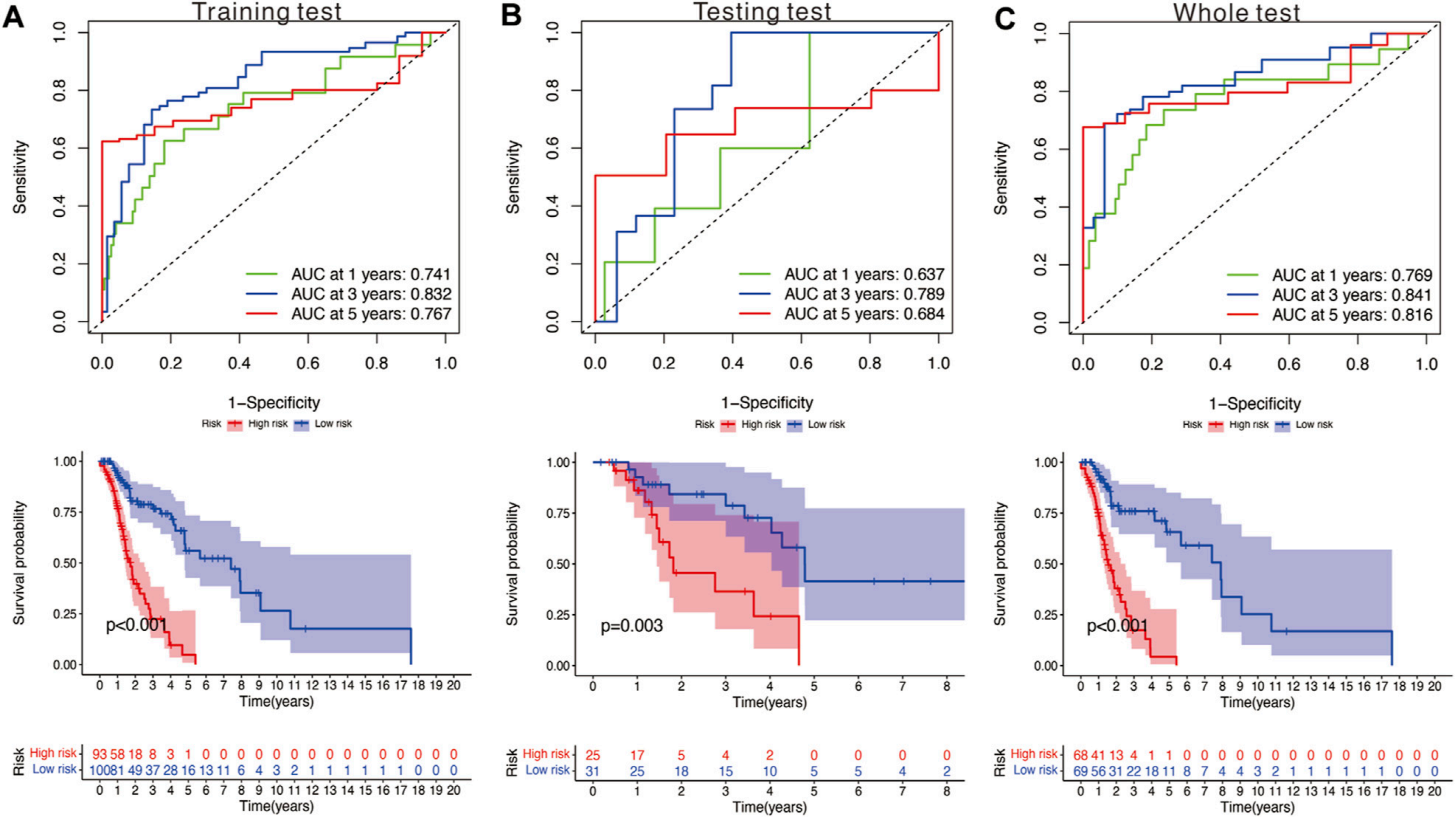
**(A)** Time‐dependent ROC analysis and survival analysis of the nine‐gene signature in the training set. **(B)** Time‐dependent ROC analysis and survival analysis of the nine‐gene signature in the testing set. **(C)** Time‐dependent ROC analysis and survival analysis of the nine‐gene signature in the whole set.

We utilized the time-dependent ROC analysis and Kaplan–Meier analysis to verify the prognostic value of the risk model. In the training set, the AUC (area under the ROC curve) values for predicting the 1-, 3-, and 5-year OS were 0.741, 0.832, and 0.767, respectively (Figure 5A). In the testing set, the AUC values for predicting the 1-, 3-, and 5-year OS were 0.637, 0.789, and 0.684, respectively (Figure 5B). In the whole set, the AUC values were 0.769, 0.841, and 0.816, respectively (Figure 5C). Moreover, HNSCC patients in the high-risk group showed significantly poorer OS than those in the low-risk group (training test: *p* < 0.001; testing set: *p* = 0.003; and whole set: *p* < 0.001).

### Development and validation of a predictive nomogram

**FIGURE 6:**
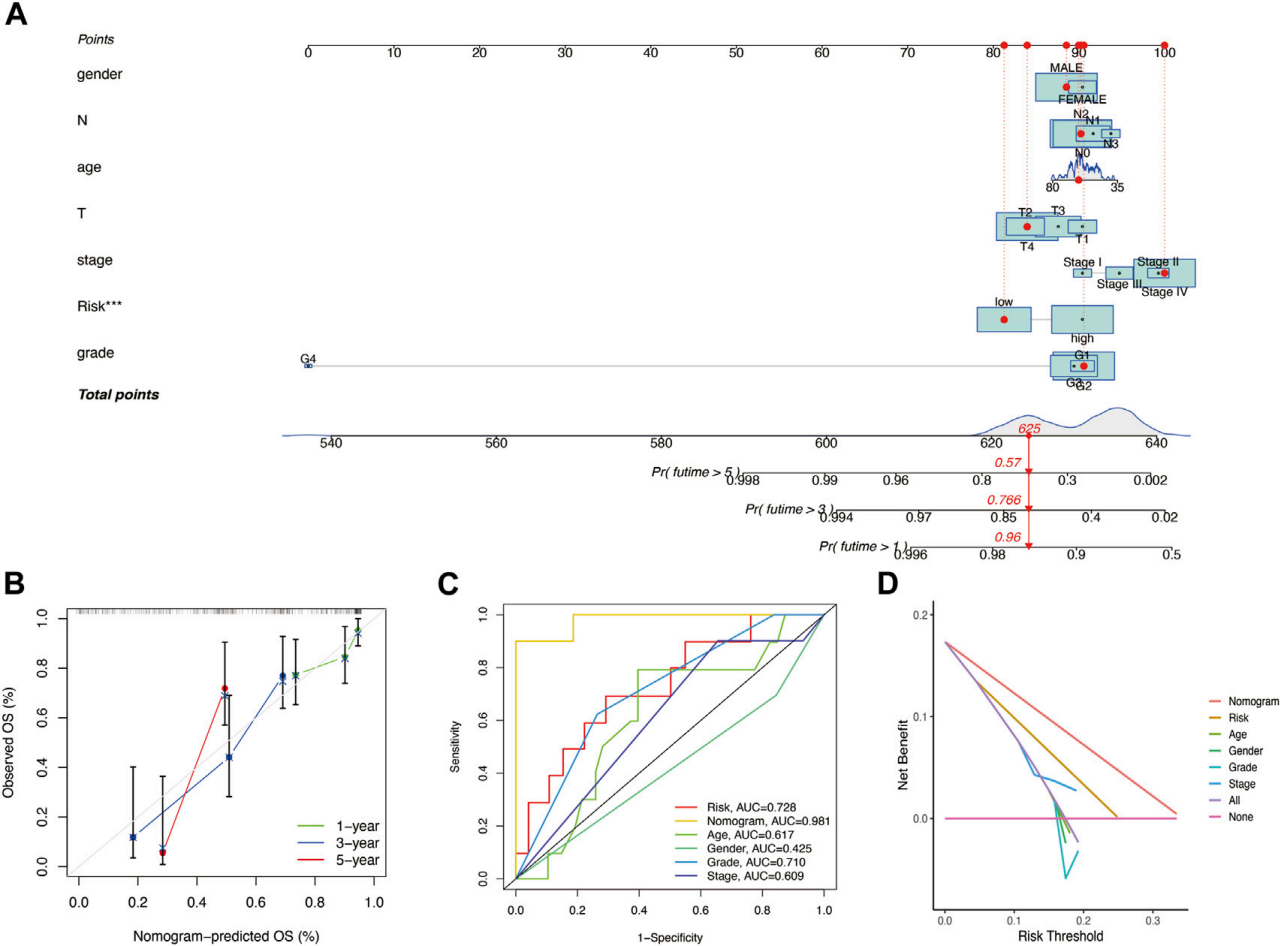
**(A)** Nomogram for predicting the OS of HNSCC patients. For each patient, lines are drawn downward to assess the points received from the seven prognostic factors in the nomogram. The sum of these points is shown on the “Total points” axis. A line is drawn downward to determine the 1‐, 3‐, and 5‐year OS of HNSCC patients. **(B)** Calibration plot for the internal validation of the nomogram. The *Y*‐axis exhibits actual survival. The *X*‐axis exhibits nomogram‐estimated survival. The dotted line (45° diagonal line) signifies full agreement between actual and observed probabilities. **(C)** Comparison of the AUC values of the nomogram and risk score, age, gender, grade, and stage. **(D)** Clinical net benefit of the nomogram, risk score, and other clinical features (including age, gender, grade, and stage).

Seven independent prognostic factors (gender, N, age, T, stage, risk score, and grade) were used to construct a nomogram for predicting the 1-, 3-, and 5-year OS for patients with HNSCC (Figure 6A). The *X*-axis indicates the nomogram’s estimated survival probability, while the *Y*-axis denotes the actual survival probability. The dotted line (45° diagonal line) between the calibration curves indicates full agreement between the actual probability and the observed probability. The calibration curves showed that the nomogram accurately predicted the 1-, 3-, and 5-year OS (Figure 6B). The estimated probability ROC curves were utilized to evaluate the sensitivity and specificity of the nine-gene signature and in predicting the survival. Among other prognostic factors (risk score, age, grade, gender, and stage), the constructed nomogram had the highest AUC value (0.981). The AUC values of the risk score, age, gender, grade, and stage were 0.728, 0.617, 0.425, 0.710, and 0.609, respectively (Figure 6C). Analysis of the nomogram’s clinical net benefit using the DCA test revealed that the model combining other independent prognostic factors had the best clinical net benefit when compared with any individual feature (Figure 6D).

### Gene set enrichment analysis

**FIGURE 7:**
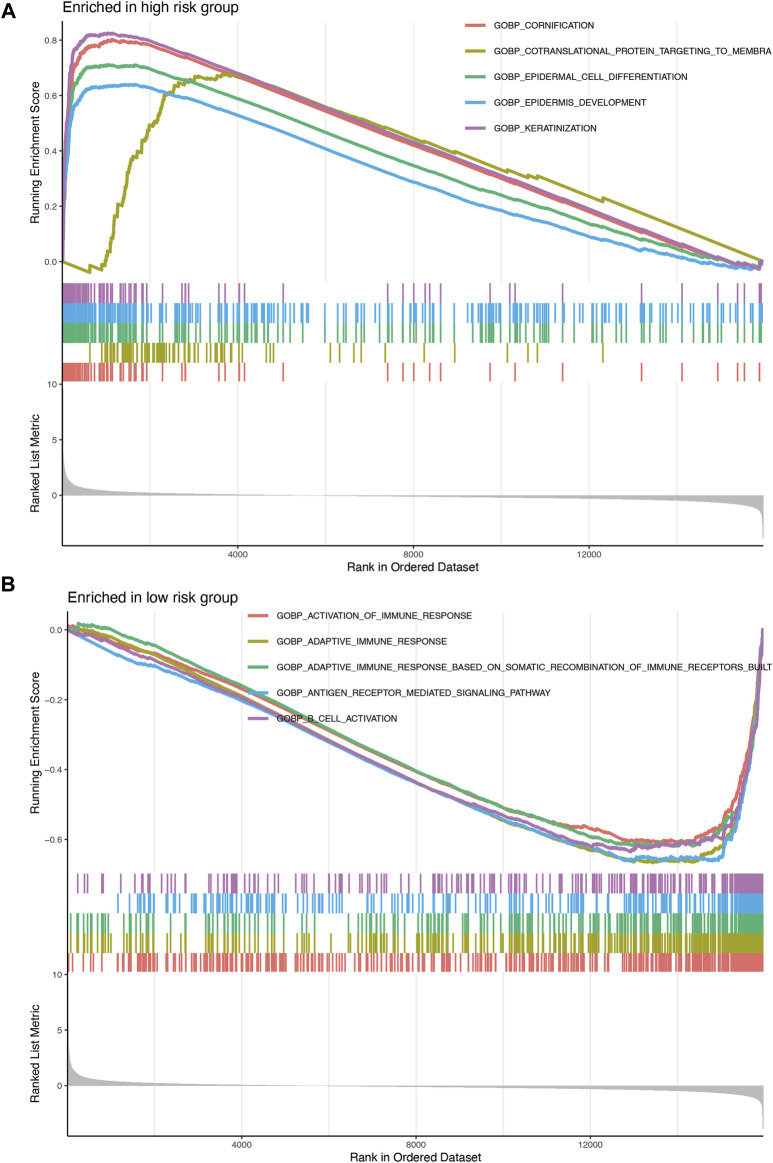
**(A)** Enrichment plots showing epidermis development (blue), epidermal cell differentiation (green), cornification (red), and keratinization (purple) gene enrichment in the high-risk group. **(B)** Enrichment plots showing the activation of immune response (red), adaptive immune response (orange), adaptive immune response based on somatic recombination of immune receptors built (green), B-cell activation (purple), and antigen receptor-mediated signaling pathway (blue) enrichment in the low-risk group.

The results of the GSEA of the nine-gene signature (Figure 7) revealed that oncological signatures (cornification, epiderma cell differentiation, epidermis development, and keratinization) were enriched in the high-risk group, whereas immune-related cascades, consisting of immune response activation, B cell activation, adaptive immune response, adaptive immune response based on somatic recombination of immune receptors built, and antigen receptor-mediated signaling cascade, were enriched in the low-risk group.

### Differentiation of the prognostic performance of the signature

**FIGURE 8:**
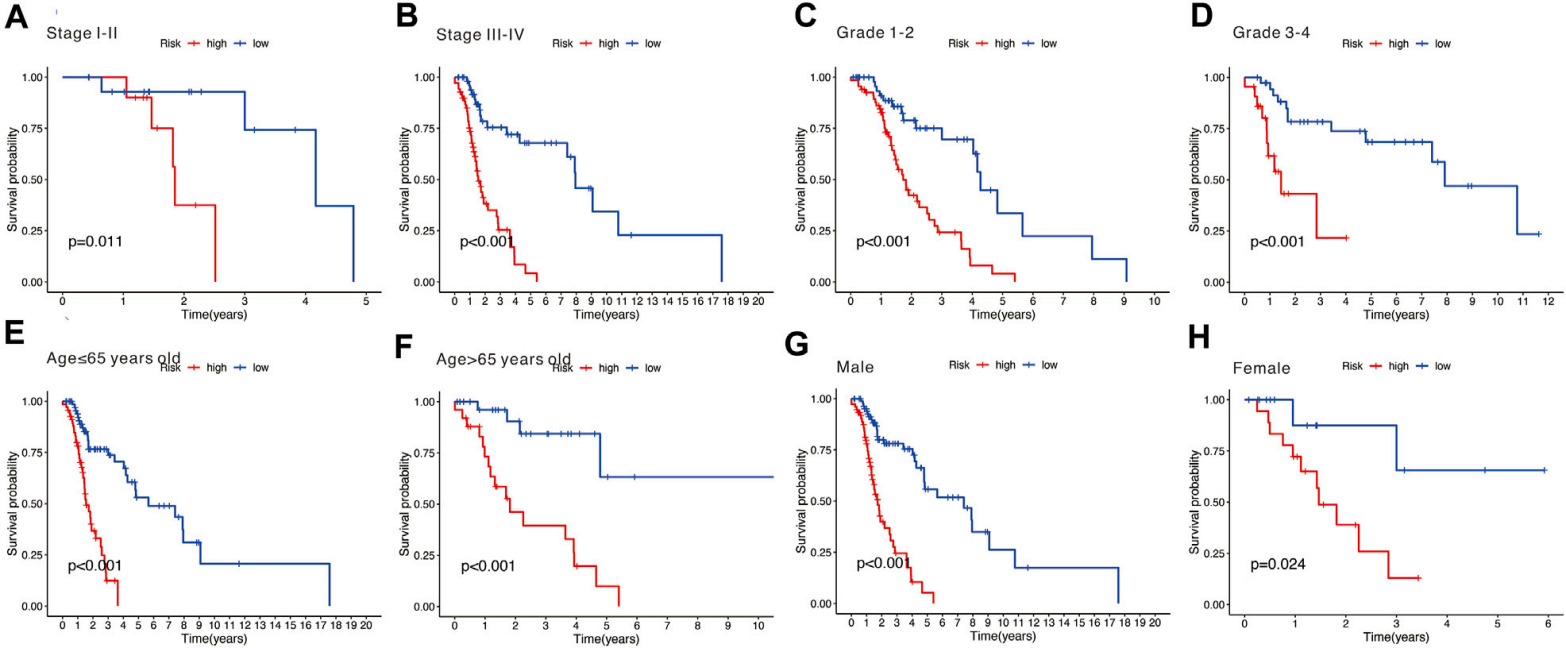
**(A,B)** Subgroup analysis stratified by different tumor stages (stages I–II and stages III–IV). **(C,D)** Subgroup analysis stratified by different tumor grades (grades 1–2 and grades 3–4). **(E,F)** Subgroup analysis stratified by different age (over 65 and under 65 years). **(G,H)** Subgroup analysis stratified by different genders (male and female).

Stratification survival analysis was performed to assess the predictive performance of the nine-gene signature in multiple HNSCC subtypes. Subgroup analysis, including stage (Figures 8A,B), grade (Figures 8C,D), age (Figures 8E,F), and gender (Figures 8G,H), was conducted to evaluate the different performances of the nine-gene signature. The results revealed that the high-risk group was associated with poorer OS and significantly differentiated the low-risk group into different subgroups (all *p* < 0.05).

### External validation of the nine-gene signature

**FIGURE 9:**
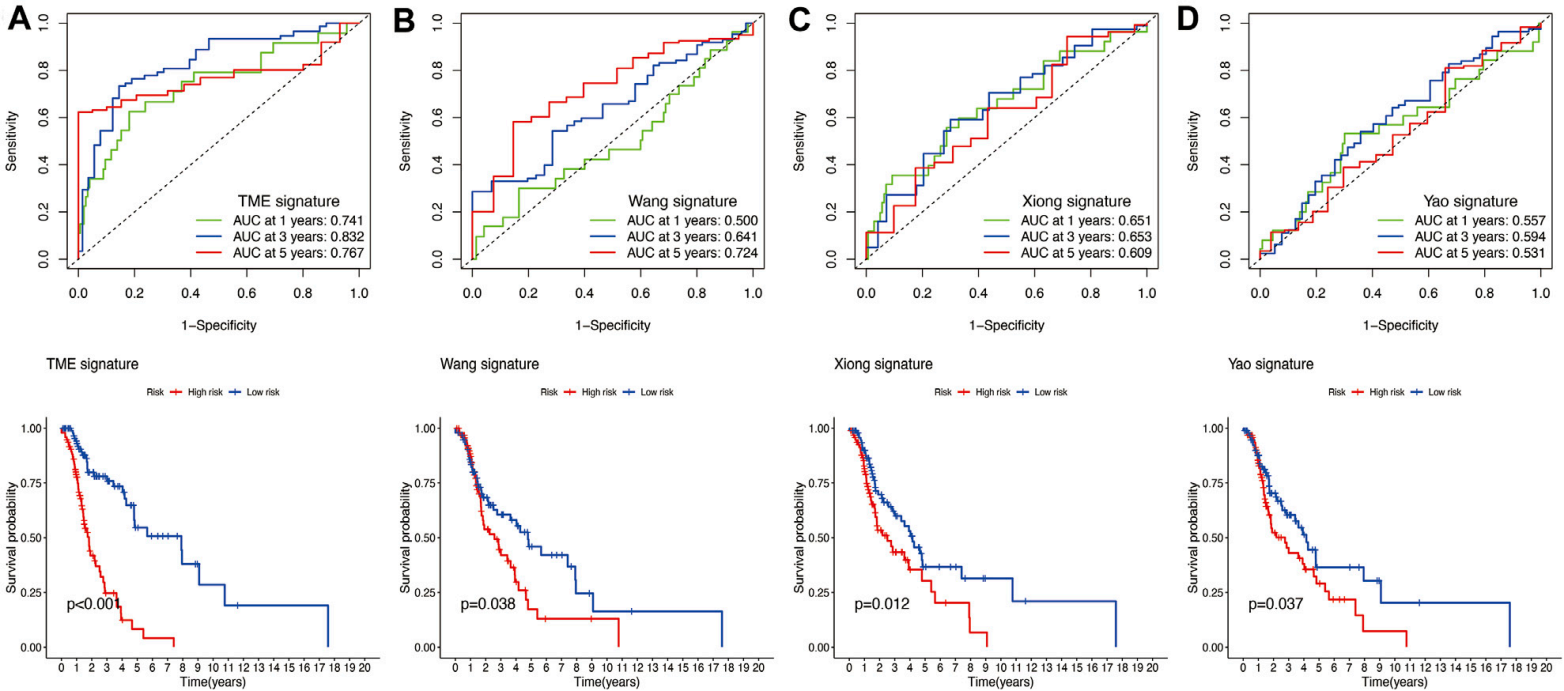
**(A)** Time‐dependent ROC analysis and survival analysis of the nine-gene signature (TME signature). **(B–D)** Time‐dependent ROC analysis and survival analysis of three-gene signatures established by other researchers.

**FIGURE 10:**
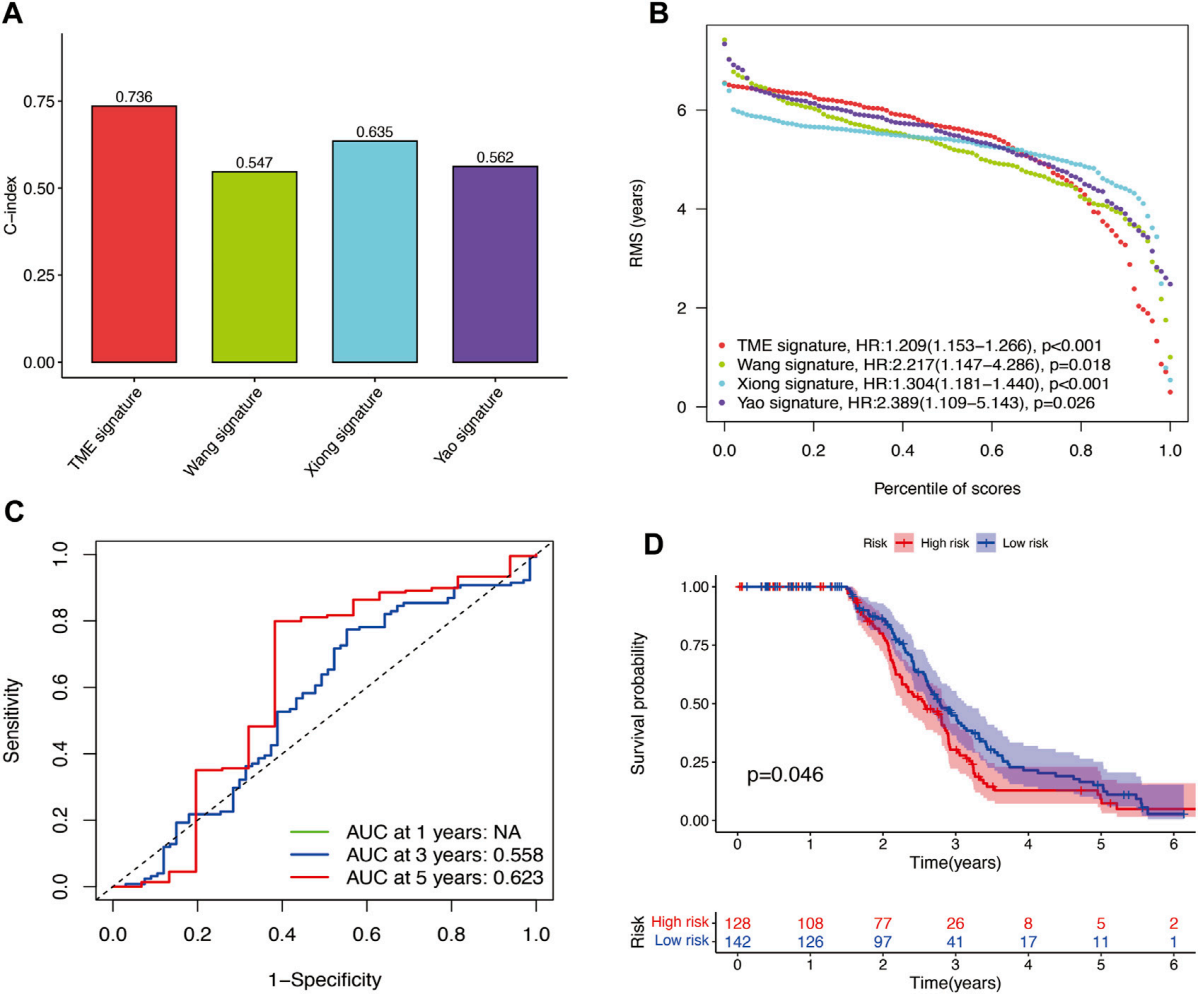
**(A–B)** C-index results and percentile of scores of the four different signatures. **(C)** AUC values for predicting 1-, 3-, and 5-year OS for patients in the GSE16076 dataset. **(D)** Survival analysis for patients in the GSE16076 dataset.

The results of time-independent ROC analysis and survival analysis indicated that the nine-gene signature had a robust and reliable performance in predicting OS. The AUC values of the nine-gene signature (TME signature) for predicting 1-, 3-, and 5-year OS were 0.741, 0.832, and 0.767, respectively (Figure 9A). Each previous gene signature was validated as a reliable prognostic model (Figures 9B–D). The results of the C-index analysis and RMS values demonstrated that the nine-gene signature was superior to other signatures in predicting the OS of HNSCC patients (Figures 10A,B). External validation of the nine-gene signature was performed using the GSE16076 dataset from the GEO database. The AUC values for predicting 1-, 3-, and 5-year OS were unknown, 0.558, and 0.623, respectively (Figure 10C). The validation of the GSE16076 dataset revealed that patients in the high-HNSCC risk group had a lower 5-year OS than those in the low-HNSCC risk group (*p* = 0.046), which was consistent with the findings from TCGA cohort (Figure 10D). Altogether, these results demonstrated that the nine-gene signature effectively predicted the survival of HNSCC patients.

### Correlation analysis

**FIGURE 11:**
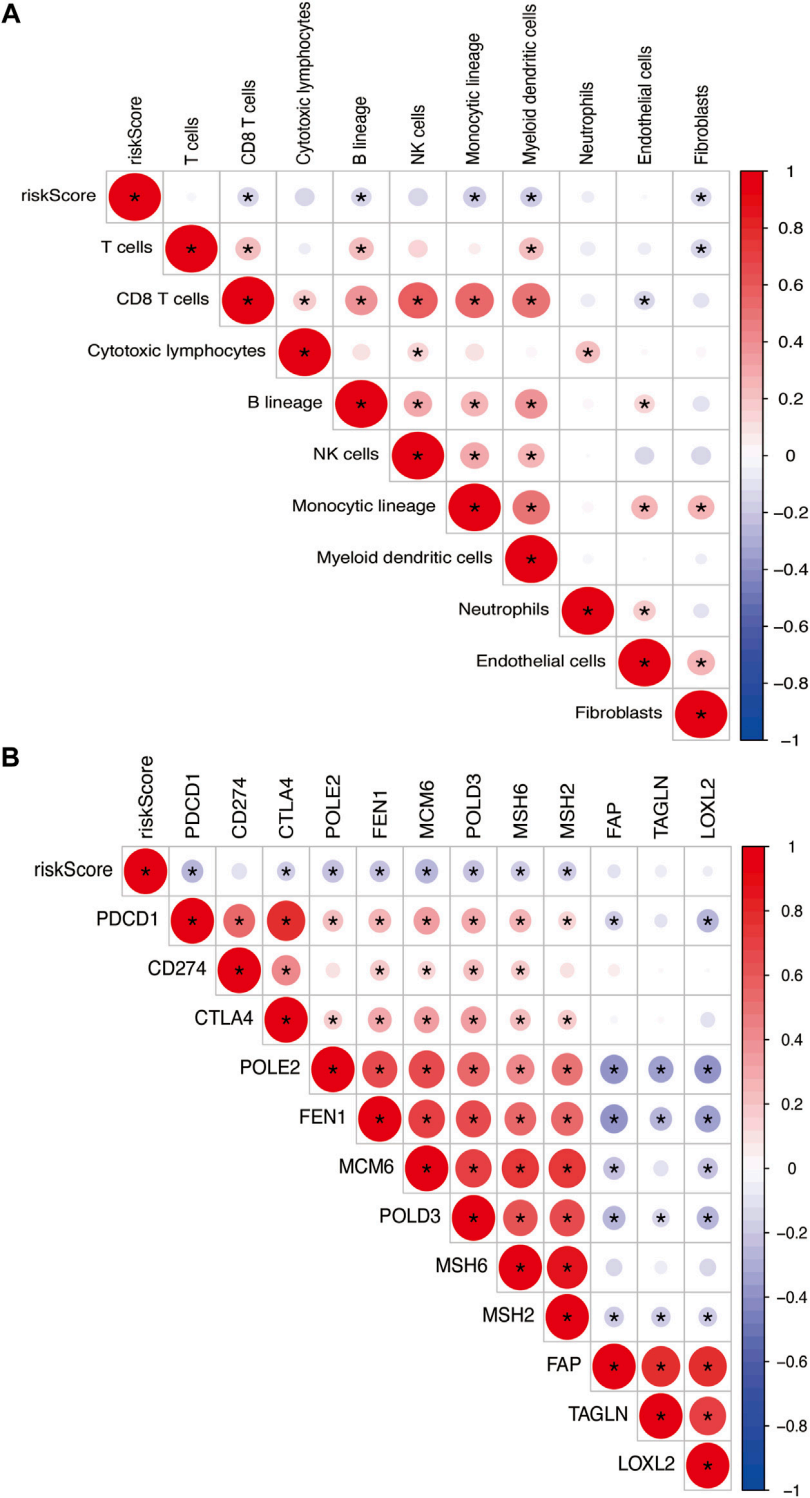
**(A)** Heatmap showing the correlation between risk score and immune cells. **(B)** Spearman correlation analysis of TME-related genes in PTC. The number on the right vertical axis represents the Spearman correlation coefficient between two genes. The black asterisk (*) inside the circle indicates *p* < 0.05. Red indicates positive correlation. Blue indicates negative correlation. The stronger the correlation, the larger the circle and the deeper the color.

The risk model was negatively correlated with CD8^+^ T cells, B lineage, monocytic lineage, myeloid dendritic cells, and fibroblasts (Figure 11A). In addition, the relationship between the risk model and these genes, such as PDCD1, CTLA4, POLE2, FEN1, MCM6, POLD3, MSH6, and MSH2, were negatively correlated (Figure 11B).

## Discussion

HNSCC is the sixth most common cancer globally, with 890,000 new cases and 450,000 deaths recorded in 2018 (Bray et al., 2018; Ferlay et al., 2019). HNSCC is characterized by early metastasis, especially lymphatic metastasis, which results in poor prognosis (Evans et al., 2019). Several studies have investigated the ability of various factors, such as TNM staging, age, gender, grade, P53 mutations, and HPV negativity, to predict HNSCC prognosis. Clinically, HNSCC is difficult to treat because of its high heterogeneity. Thus, reliable prognostic tools are urgently needed to promote accurate prediction of HNSCC outcomes. Recent studies have indicated that signatures based on aberrantly expressed genes can predict cancer prognosis (Cohen et al., 2019).

In the present study, we developed a nine-gene signature consisting of GTSE1, LRRN4CL, CRYAB, SHOX2, ASNS, KRT23, ANGPT2, HOXA9, and CARD11 for predicting HNSCC prognosis. Internal validation based on TCGA database and external validation based on the GEO database were conducted and showed that the prognostic model is robust and reliable.

Through the tumor-suppressive protein p53, GTSE1 has been implicated in the pathogenesis of several malignant tumors. Previous research has proved its negative regulation in malignant tumors (Lai et al., 2021). Moreover, GTSE1 has been linked to the development of chemoresistance in osteosarcoma (Xie et al., 2021) and hepatocellular carcinoma (Li et al., 2021). To date, the role of GTSE1 in HNSCC has not been fully clarified. LRRN4CL is a cytotoxicity-associated gene associated with CD8^+^ T-cell infiltration. Upregulation of LRRN4CL expression on cell surfaces correlates with increased pulmonary metastases in mice (van der Weyden et al., 2021). However, the mechanism of LRRN4CL upregulation in HNSCC was not clear. CRYAB, a member of the small heat shock protein family, can regulate several cellular processes, including apoptosis, inflammation, and oxidative stress (Zhang et al., 2019). Under hypoxic conditions, CRYAB upregulation was shown to improve the survival of HNSCC cells (van de Schootbrugge et al., 2014). The relationship between CRYAB expression and HNSCC survival outcome is indistinct (Boslooper et al., 2008). Methylated SHOX2 in circulating cell-free DNA correlates with the tumor stage and prognosis (Franzen et al., 2020). There is evidence that SHOX2 can predict the occurrence and prognosis of HNSCC (Bergheim et al., 2018). It has been reported that ASNS transforms aspartate and glutamine to asparagine and glutamate in an ATP-dependent manner, respectively. The activity of human ASNS is influenced by cellular stress (Lomelino et al., 2017). The high expression of ASNS promotes growth, metastasis, and chemoresistance in neoplastic cells. In contrast, the low expression of ASNS impaired metabolic function in specified cancer models (Chiu et al., 2019). ASNS is also an important predictor of HNSCC prognosis (Mai et al., 2021). Loss of KRT23 affects the cell cycle, DNA replication, recombination, and repair (Birkenkamp-Demtröder et al., 2013). Evidence from previous studies has indicated that KRT23 influences the proliferation, migration, and prognosis of cancer (Odena et al., 2016; Zhang et al., 2017; Chen et al., 2021). ANGPT2 is a ligand for TIE1–TIE2 signaling involved in the development and maintenance of blood and lymphatic vessels (Smeland et al., 2021). In this study, we found that ANGPT2 regulates physiological and pathological angiogenesis in HNSCC by modulating vasodilation, microvascular permeability, and vasoconstriction (Xu et al., 2019). ANGPT2-deficient mice show abnormalities in blood and lymphatic vasculature and deficits leukocyte mobilization to inflammation sites (Fiedler et al., 2006). ANGPT2 is associated with shorter OS in HNSCC (Arends et al., 2021). HOXA9, a homeotic transcription factor (Lambert et al., 2019), is upregulated in HNSCC tissues and cells. HOXA9 knockdown inhibits cell proliferation, migration, invasion, and chemoresistance (Zhou et al., 2019; Sun et al., 2020). CARD11, a key member of the protein arginine methyltransferase (PRMT) family, encodes an adaptor protein that expresses dominant-negative (Guo et al., 2020). CARD11 gain-of-function variants may cause various immunodeficiencies (Meitlis et al., 2020). Mounting evidence indicates that CARM1 is associated with carcinoma metastasis (Sharma et al., 2017; Cai et al., 2019; Hu et al., 2020). CARM1-silencing in oral squamous cell carcinoma cells effectively suppresses tumor invasion (Lyu et al., 2022).

To our knowledge, the use of nine-gene signature for predicting the prognosis of HNSCC has not been reported before. The risk score for each of the nine prognostic genes was based on the gene expression profile but not somatic mutations or methylation status. This signature can be applied in many clinical centers because it is affordable and eliminates the requirement for whole-genome sequencing. Although our results relied on an open-access online TCGA database, without further validation of conventional prediction methods, we chose the GSM16076 dataset to validate our results. The results of the time-independent ROC analysis and survival analysis indicated that the nine-gene signature is reliable and stable. Moreover, the nomogram showed better performance than conventional clinical parameters, especially in predicting short-term (1-year or 3-year) survival. Thus, our signature can be used to guide clinical decisions regarding HNSCC treatment.

## Conclusion

In conclusion, we established and verified a TME-related nine-gene signature and nomogram, which might have the potential to act as new therapeutic targets for HNSCC patients.

## Data Availability

The original contributions presented in the study are included in the article/Supplementary Materials; further inquiries can be directed to the corresponding author.
